# PF-127 hydrogel plus sodium ascorbyl phosphate improves Wharton’s jelly mesenchymal stem cell-mediated skin wound healing in mice

**DOI:** 10.1186/s13287-020-01638-2

**Published:** 2020-04-03

**Authors:** Qingzha Deng, Sunxing Huang, Jinkun Wen, Yiren Jiao, Xiaohu Su, Guang Shi, Junjiu Huang

**Affiliations:** 1 Key Laboratory of Reproductive Medicine of Guangdong Province, The First Affiliated Hospital and School of Life Sciences, Sun Yat-sen University, Guangzhou, 510275 China; 2 MOE Key Laboratory of Gene Function and Regulation, State Key Laboratory of Biocontrol, School of Life Sciences, Sun Yat-sen University, Guangzhou, 510275 China; 3 Jiangmen Central Hospital, Affiliated Jiangmen Hospital of Sun Yat-sen University, Guangzhou, 510150 China

**Keywords:** PF-127 hydrogel, Sodium ascorbyl phosphate, Wharton’s jelly mesenchymal stem cells, Skin wound, Cell engraftment

## Abstract

**Background:**

Factors such as poor engraftment, retention, and survival of the transplanted stem cells are deemed to limit their therapeutic efficacy for wound regeneration. Hence, it is necessary to explore these issues in order to resolve them. In this study, we aim to investigate the role of Pluronic F-127 (PF-127) hydrogel plus antioxidant sodium ascorbyl phosphate (SAP) in enhancing Wharton’s jelly mesenchymal stem cell (WJMSC)-mediated effectiveness on full-thickness skin wound healing in mice.

**Methods:**

First, the cytotoxicity of PF-127 and the biological effect of SAP on the survival of WJMSCs were tested in vitro using cell viability and proliferation assays. Next, a cell suspension containing WJMSCs, PF-127, and SAP was topically administered onto an 8-mm diameter excisional full-thickness wound bed. Eight days after transplantation, the mice were sacrificed and the skin tissue was excised for histological and immunohistochemical analysis. Finally, in vivo distribution of transplanted WJMSCs was traced to investigate cell engraftment and the potential therapeutic mechanism.

**Results:**

PF-127 was found to be cytotoxic to WJMSCs while SAP significantly improved the survival of PF-127-embedded WJMSCs. When this combination was topically transplanted onto the wound bed, wound healing was facilitated and dermis regeneration was achieved on the 8th day after surgery, as evidenced by an increase in dermal thickness, newly developed hair follicles, and collagen fiber deposition accompanied by a reduction in scar width. Further, immunohistochemical analysis demonstrated a higher number of anti-inflammatory M2 macrophages, proliferating cells, and newly formed blood vessels in the WJMSCs/PF-127/SAP group relative to all other groups. In addition, in vivo tracking results revealed a highly enhanced engraftment of WJMSCs accumulated in the dermis in the WJMSCs/PF-127/SAP group.

**Conclusions:**

SAP significantly improves the survival of WJMSCs in PF-127 encapsulation. Further, PF-127 plus SAP is an effective combination that enhances WJMSC engraftment in the dermis, which then promotes full-thickness wound healing through potential M2 macrophage formation and angiogenesis.

## Introduction

Destruction of structural and functional integrity of normal skin caused by traumatic injuries can lead to wound formation [1]. In general, minor injuries which do not affect the dermal layer, also referred to as superficial wounds, can be completely self-healing, while major injuries involving deeper dermal layers defined as full-thickness wounds heal slowly and are susceptible to the development of abnormal fibrotic scars or chronic inflammation. For large cutaneous lesions, traditional therapies such as debridement, addition of growth factors, or dressing of the wounds have been used in clinical practice [2–4]. Nonetheless, delayed healing, excessive fibrotic healing, and development of chronic inflammation remain great challenges.

Due to the recent advances in stem cell research, stem cell-based therapy provides a potential alternative strategy for wound regeneration [5]. Several kinds of stem cells have been utilized for this purpose; moreover, Wharton’s jelly-derived mesenchymal stem cells (WJMSCs) are considered to be one of the most promising cell sources of regeneration medicine due to their fewer ethical issues and safety concerns when compared to the tumorigenicity and ethical controversy of ESCs and the tumor formation and immunogenicity potential of iPSCs [6–8]. Further, WJMSCs isolated from fetal tissue have several unique beneficial properties including easy isolation, higher proliferation capacity, and potentially strong immunomodulatory effects [9]. Previous studies have reported the beneficial effects of WJMSCs on large full-thickness cutaneous wounds in animal models [10, 11]. However, it is believed that poor engraftment, retention, and survival of the transplanted stem cells are the important barriers affecting their therapeutic efficiency on wound regeneration [12, 13]. Therefore, to maximize the therapeutic benefits, the efficacy of WJMSC-mediated large full-thickness wound therapy needs to be fully elucidated.

Tissue engineering-based strategy demonstrates that the utilization of biomaterial-based scaffolds as stem cell delivery and retention platforms can enhance the therapeutic efficiency of stem cells on wound regeneration [14–16]. Pluronic F-127 (PF-127, also known as poloxamer 407), a FDA-approved synthetic copolymer made of poly (ethylene oxide)-poly (propylene oxide)-poly (ethylene oxide), is injectable, biodegradable, and thermo-reversible [17, 18]. Recent evidences have shown the therapeutic effects of PF-127 used as a scaffold for drug delivery, extracellular vesicles, and cell encapsulation in tissue engineering [19–21]. However, it has also been reported that PF-127 encapsulation has a negative effect on cell viability [22–24]. Hence, to optimize the therapeutic efficacy of WJMSCs, improving their survival in PF-127 encapsulation is of great significance before clinical application.

Sodium ascorbyl phosphate (SAP) is a sodium salt of ascorbic acid 2-phosphate and it exhibits high stability even on long-term exposure to reactive oxygen species as well as in aqueous solutions environment when compared to vitamin C [25]. SAP is also a strong antioxidant and its biological function is similar to that of ascorbic acid, that is, removal and destruction of excess harmful reactive oxygen radicals in cells [26]. It has been reported that SAP could act as an antioxidant to protect cultured mouse skin from UVB-induced damage [27]. Further, other researchers have reported that combining a urea-cross-linked hyaluronic acid biopolymer with SAP exerts an enhanced antioxidative and anti-inflammatory activity against several kinds of inflammation-associated diseases [28, 29]. Additionally, SAP has been shown to stimulate collagen synthesis in cultures of human dermal fibroblasts, suggesting that SAP is likely to play a crucial role in the wound healing process [30].

In this study, we aimed to evaluate the biological effect of SAP on the survival of WJMSCs in PF-127 encapsulation. Additionally, a cell suspension composed of WJMSCs, PF-127, and SAP was transplanted in vivo to investigate whether this combination could enhance the efficiency of WJMSCs in full-thickness skin wound healing. Finally, in vivo distribution of transplanted WJMSCs was traced to investigate cell engraftment and the potential therapeutic mechanism.

## Materials and methods

### Animal

C57BL/6 male mice were purchased from Guangdong Medical Laboratory Animal Center (Guangzhou, China). Eight-week-old mice weighting 20–30 g were used to establish full-thickness acute wound model. All animal experiments were conducted according to the protocols approved by the Ethics Committee on Animal Care, Sun Yat-sen University, China.

### WJMSCs isolation from human umbilical cord

This study was approved by the Ethical Committee of the First Affiliated Hospital of Sun Yat-sen University. Written informed consent was obtained from a patient prior to donating the healthy umbilical cord for research. WJMSCs isolated from the human umbilical cord were performed according to protocols from previous report [31]. Briefly, the umbilical cord (UC) obtained from a normal delivery healthy donor with informed donor consent was immediately carried back to the laboratory in sterile phosphate-buffered saline (PBS) containing 100 U/mL penicillin and 100 μg/mL streptomycin (Gibco, USA). After cleanly washing the outside of UC with PBS to remove blood, the tissue was cut into smaller pieces. Each of the pieces was washed and squeezed with sterile curved tweezers to remove vessels internal blood and then cut lengthwise to remove the vessels. Finally, the remaining tissue was washed cleanly, minced into about 1–3 mm^2^ smaller pieces, and plated on 150 mm dishes supplemented with 10% fetal bovine serum (Hyclone, USA) in DMEM-F12 medium (Corning, USA) containing 1% penicillin/streptomycin to allow cells to migrate from tissue margin. The cells were subcultured to the 4th passage for all in vitro assays and in vivo transplantation.

### Flow cytometry analysis

The 1st and 4th passage WJMSCs were digested with 0.25% trypsin-EDTA (Gibco, USA) and 1 × 10^5^ cells were re-suspended in 100 μL PBS, followed by incubating with 1 μL phycoerythrin (PE) or 5 μL fluorescein isothiocyanate (FITC)-conjugated anti-human MSC-positive antibodies (CD13, 12-0138-42; CD29, 11-0299-42; CD44, 11-0441-82; CD73, 11-0739-42; CD90, 12-0909-42; CD105, 12-1057-42; HLA-ABC, 12-9983-42) or MSC-negative antibodies (CD31, 11-0319-42; CD14, 11-0149-42; CD45, 11-0452-82; HLA-DR, 11-9956-42) on ice in the dark for 30 min, respectively. Additionally, FITC-conjugated mouse IgG1 (11-4714-42) and PE-conjugated mouse IgG1 (12-4714-81) antibodies were used as isotype control. All antibodies were bought from eBioscience, England. Cytometric analysis was performed using a flow cytometer (CytoFLEX Beckman Coulter, USA) and the results were analyzed using Cell Quest Pro Software.

### Adipogenic differentiation

Adipogenesis was assessed by OriCell™ Adipogenesis Differentiation Kit (HUXUC-90031, Cyagen, China), following the manufacturer’s instructions. Briefly, WJMSCs were placed onto a 6-well plate at a density of 2 × 10^4^ cells/cm^2^. Upon the cell reaching confluence, the medium was replaced with the adipogenic induction medium for another 3 weeks induction. Finally, the cells were fixed with 4% neutral formalin solution and stained with Oil Red O working solution to detect fat droplet formation.

### Osteogenic differentiation

Osteogenesis was assessed by OriCell™ Osteogenesis Differentiation Kit (HUXUC-90021, Cyagen, China), following the manufacturer’s instructions. Briefly, WJMSCs were placed onto a 0.1% gelatin preconditioned 6-well plate at a density of 2 × 10^4^ cells/cm^2^. Upon the cell confluence reached about 60%, the medium was replaced with the osteogenic induction medium for another 3 weeks induction. Finally, the cells were fixed with 4% neutral formalin solution and stained with Alizarin Red S working solution to detect calcium deposits.

### Chondrogenic differentiation

Chondrogenesis was assessed by OriCell™ Chondrogenesis Differentiation Kit (HUXUC-90041, Cyagen, China), following the modified manufacturer’s instructions. Briefly, WJMSCs were placed onto a 0.1% gelatin preconditioned 6-well plate at a density of 2 × 10^4^ cells/cm^2^. Upon the cell reaching confluence, the medium was replaced with the chondrogenic induction medium for another 3 weeks induction. Finally, the cells were fixed with 4% neutral formalin solution and stained with Alcian blue working solution to detect proteoglycan deposits.

### PF-127 hydrogel preparation and cell encapsulation

PF-127 hydrogel preparation was performed according to previous report [32]. Briefly, 20% (w/v) PF-127 solution was dissolved in DMEM-F12 medium at 4 °C, then filtered through a 0.22 μm filter (Millipore, USA), and finally kept at 4 °C for use. For in vitro PF-127 hydrogel cytotoxicity test, WJMSCs were encapsulated within PF-127 solution with 0, 400, or 800 μM SAP (49752, Sigma, USA) in 37 °C incubator (5% CO_2_) for 5 min to make gel formation. Finally, a further complete culture medium was added over the gel and transferred back to the incubator for another 24 h co-cultivation.

### CCK cell viability assay

Cell Counting Kit-8 (CK04, Dojindo, Japan) was used to test cell viability in PF-127 hydrogel encapsulation. Briefly, the WJMSCs were seeded at a density of 1 × 10^4^ cells/100 μL with different encapsulation conditions in 96-well plate for 24 h. Cell viability was evaluated by adding 10% CCK reagent to each well and incubated for 2 h before measuring the absorbance at 450 nm using microplate reader (VICTOR™ X5, PerkinElmer, USA).

### Cell live and dead assay

Cell survival in PF-127 hydrogel encapsulation was assessed by using Live/Dead™ Cell Imaging Kit (R37601, Invitrogen, USA) following the manufacturer’s instructions. Briefly, the WJMSCs were seeded at a density of 5 × 10^4^ cells/500 μL with different encapsulation conditions in 12-well plate for 24 h. Then, A and B suspension solutions were mixed and added to each well to incubate for 15 min in dark before detecting cell survival using an inverted fluorescence microscope (Axio observer A1, Zeiss, Germany).

### Cell proliferation assay

Cell proliferation in PF-127 hydrogel encapsulation was investigated by using Cell Light™ EdU Kit (C10310, Ribo Biotech Company, China) according to the manufacturer’s instructions. Briefly, the WJMSCs were seeded at a density of 2 × 10^4^ cells/500 μL with different encapsulation conditions in 12-well plate for 24 h. Then, 50 μM EdU was added to each well for another 2 h followed by 4% PFA fixation, 0.5% TritonX-100 permeabilization, and finally Apollo and DAPI staining. Fluorescent images were obtained by using an inverted fluorescence microscope.

### Cell wound scratch assay

Cell migration in PF-127 hydrogel encapsulation was investigated. Briefly, the WJMSCs were seeded at a density of 1 × 10^5^ cells with different encapsulation conditions in 6-well plate for 24 h. After the cells reached confluence, a straight line was scratched with 1 mL tip and imaged at the indicated time point. Relative migration rate was calculated by Image J software (National Institute of Health, USA) as follows: relative migration rate (%) = Mf/Mi × 100%, where Mi is the initial migration distance at 0 h while Mf is the final migration distance at 12 h or 24 h, respectively.

### Skin wound model establishment and in vivo transplantation

To establish a major full-thickness skin wound model, the mice were anesthetized by intraperitoneal injection of 50 mg/kg sodium pentobarbital (Sigma-Aldrich). After removing the hair with depilatory wax, two full-thickness wounds with a diameter of 8 mm were created with a biopsy punch on the dorsum. For in vivo transplantation, the mice were randomly divided into eight groups. Fifty microliters of PF-127 solution encapsulated with a dose of 1 × 10^6^ WJMSCs and 400 μM SAP or other combinations were topically placed onto the wound site and then covered with IV3000 transparent dressing to avoid detachment and infection. Mice were kept in individual cage and observed daily during the experiment. At the indicated time point, the residual skin wound was imaged. Unhealed wound area was calculated by Image J software as follows: unhealed wound area (%) = Wr/Wi × 100%, where Wi is the initial wound area at day 0 while Wr is the residual wound area at day 5 and day 8 post-transplantation.

### Histological analysis

At the eighth day after surgery, the mice were sacrificed for histological analysis. Briefly, the wound bed together with surrounding tissue were excised and underwent following standard procedures including 4% paraformaldehyde fixation, gradual dehydration, and paraffin embedding. The embedded tissues were then sliced into 5-μm-thick sections in the direction of hair flow, which were further stained with a Hematoxylin and Eosin Staining Kit (G1120, Solarbio, China) to detect morphological changes and Masson Trichrome Staining Kit (D026, Nanjing Jiancheng Bioengineering Institute, China) to evaluate the synthesis of collagen. Image J software was used to measure the thickness of the dermis, the scar width, and the total number of hair follicles.

### Lentiviral vector construction and transduction

The CDS region of EGFP PCR product was cloned into pENTR by the way of double digestion and ligation reactions. The resulting vector, termed pENTR-EGFP, was then recombined into the pLenti-CAG-Dest-IRES-Puro vector using a recognized LR recombination reaction method following the manufacturer’s instructions (11791100, Gateway™ LR Clonase™ II Enzyme mix, Invitrogen). The final lentiviral expression vector was designated pLenti-CAG-EGFP-IRES-Puro.

Lentiviruses were prepared by transient co-transfection of HEK293T cells with the targeted vector, pLenti-CAG-EGFP-IRES-Puro, and lentiviral packaging vectors mix, pMD2.G and psPAX2 by using PEI (306185, Sigma, USA). Three days after transfection, supernatants containing viral particles were harvested, filtered through polyether sulfone membranes (pore size, 0.45 mm), and tittered.

For lentiviral transduction, a density of 50% P4 WJMSCs was transduced with lentivirus particles and 4 μg/mL polybrene (TR1003, Sigma, USA). Seventy-two hours after transduction, 1 mg/mL was added to the medium for screening 3 to 4 days. After that, retrieved the complete medium for culturing continuously until to the cell confluence of 90%. Finally, fluorescence image and western blotting were performed to verify the establishment of a stably EGFP-overexpressing cell line.

### In vivo tracking of transplanted WJMSCs

To track the distribution of transplanted MSC^EGFP^, MSC^EGFP^/PF-127, MSC^EGFP^/400 μM SAP, or MSC^EGFP^/PF-127/400 μM SAP in vivo, the samples were collected from the skin tissue at 24 h and 72 h post-transplantation. Cryosections were prepared and counterstained with DAPI for 5 min. After that, the sections were observed under a fluorescence microscope.

### Immunohistochemical analysis

Sections were undergone antigen retrieval with sodium citrate buffer and blocked by 3% goat serum (16210064, Gibco, USA) for 1 h. Then primary antibodies (Rabbit anti-CD31, 1:200, GTX130274, Gene Tex; Rabbit anti-Ki-67, 1:300, 12202 T, Cell Signaling Technology; Rabbit anti-CD163, 1:300, ab182422, Abcam) diluted in blocking buffer were used to incubate at 4 °C overnight. After washing with PBS, the Cell and Tissue Staining Kit (CTS005, Anti-Rabbit HRP-DAB System, R&D Systems) was used to detect the positive staining area. Images were taken by a microscope and analyzed by Image J software.

### Statistical analysis

All the data were demonstrated as mean ±standard error of mean (SEM). One-way analysis of variance (ANOVA) was used to analyze the multiple group comparisons followed by Tukey’s post test using GraphPad Prism 5.0 software. *p* < 0.05 was considered statistically significant (*p < 0.05, **p < 0.01, ***p < 0.001; ns, no significance).

## Results

### Isolation and identification of Wharton’s jelly-derived mesenchymal stem cells from human umbilical cord

**Fig. 1:**
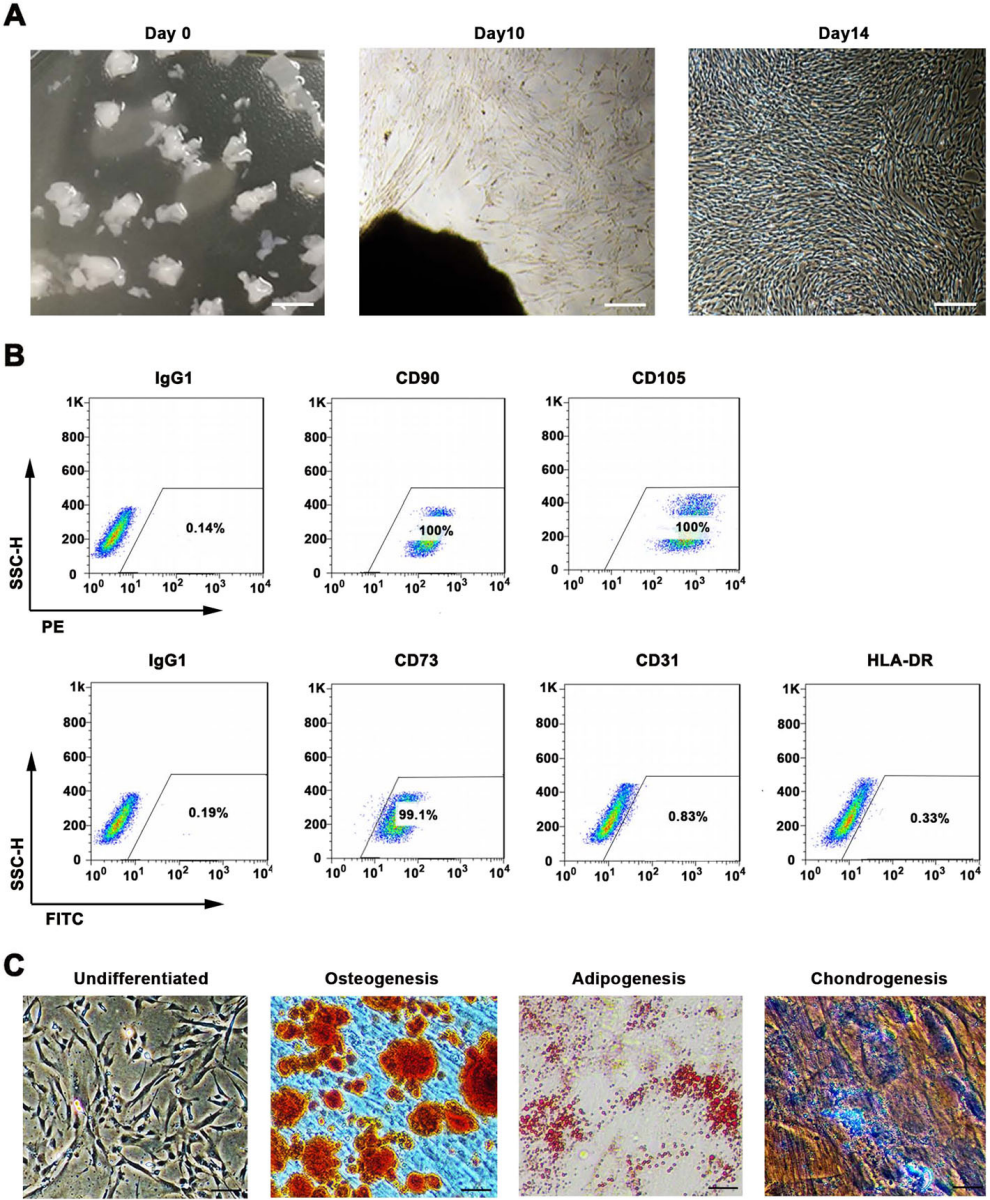
WJMSCs isolation and characterization. **a** Primary cell isolation procedure from Wharton’s jelly tissue. The migrated cells exhibited typical fibroblast-like morphology. Scale bar, 500 μm. **b** Flow cytometry analysis of P4 cells using mesenchymal stem cell markers (CD90, CD105, CD73), endothelial cell marker (CD31), and MHC class II protein HLA-DR. Isotypic antibodies (IgG1-PE and IgG1-FITC) were used as negative controls. **c** Representative stained images show that the fourth passage WJMSCs could differentiate into osteocytes (Alizarin Red S), adipocytes (Oil Red O), and chondrocytes (Alcian blue). Scale bar, 100 μm

For isolation of MSCs from Wharton’s jelly, we used the explant culture method, because the cell proliferation capacity and cell viability were higher by this method compared to the enzymatic digestion method [33]. As shown in Fig. 1a, 2 weeks post isolation, the migratory cells exhibited typical fibroblast-like morphology and plastic-adherent characteristic. Fluorescence associated cell sorting (FACS) and differentiation assays were further performed to confirm whether these cells were MSCs. FACS analysis revealed that over 97% of the 1st passage cells were positive for MSCspecific markers, such as HLA-ABC, CD105, CD13, CD29, CD44, and CD73, and the expression of endothelial cell marker CD31, hematopoietic cells markers CD14 and CD45, and major histocompatibility complex (MHC) class II protein HLA-DR was low in these cells (Fig. S1A). In addition, more than 99% of the 4th passage cells continued expressing MSC surface markers including CD90 (100%), CD105 (100%), and CD73 (99.1%), while less than 1% of cells expressed endothelial cell marker CD31 (0.83%) and major histocompatibility complex (MHC) class II protein HLA-DR (0.33%) (Fig. 1b). In addition, in vitro differentiation assay demonstrated that these cells were able to differentiate into osteocytes, adipocytes, and chondrocytes (Fig. 1c). Collectively, the cells isolated from Wharton’s jelly accorded with the minimal criteria set by the International Society for Cellular Therapy (ISCT) for defining MSCs [34], and therefore, the isolated cells were defined as WJMSCs.

### SAP improves WJMSCs survival in PF-127 hydrogel encapsulation

**Fig. 2:**
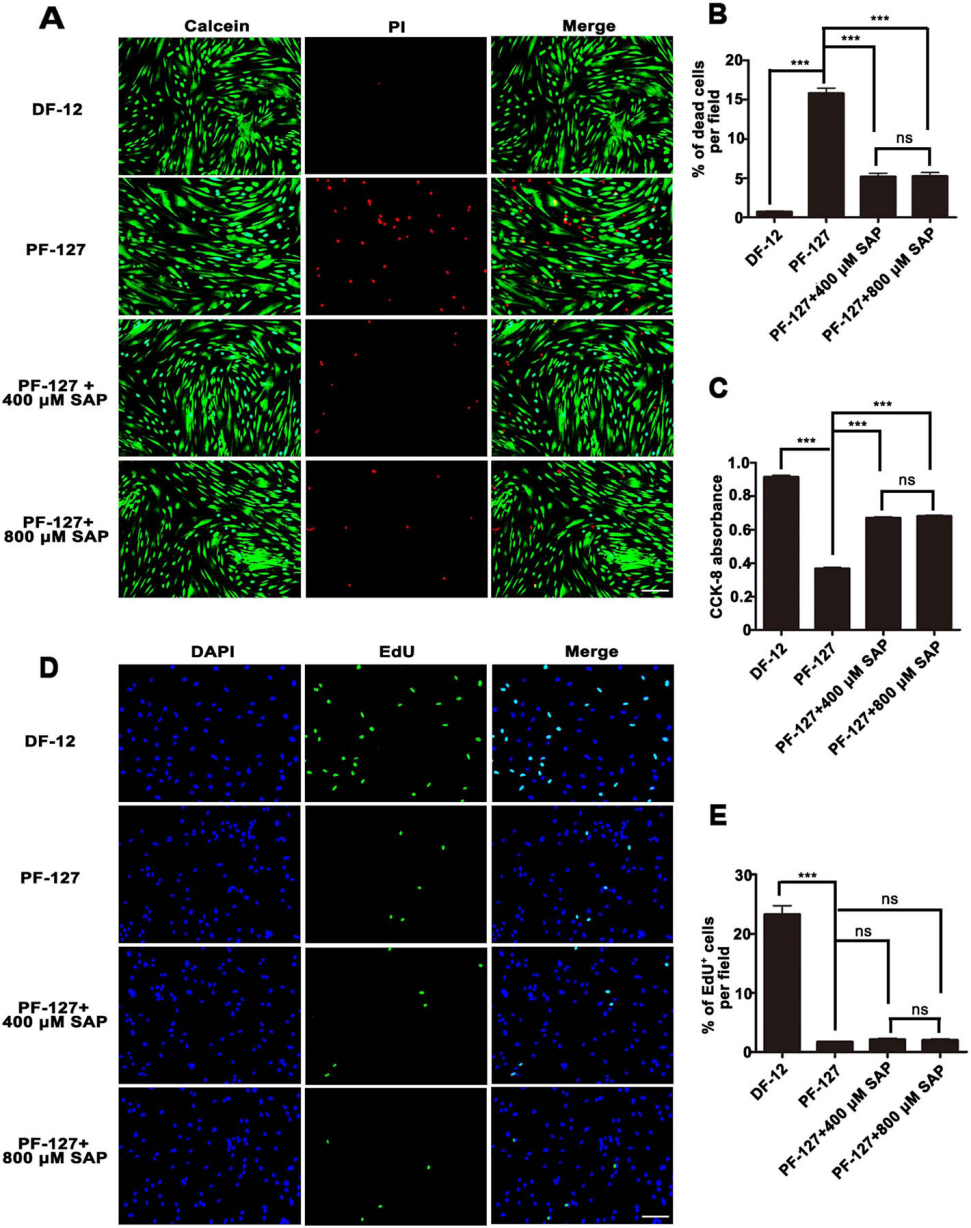
SAP improves Wharton’s jelly mesenchymal stem cell survival in PF-127 encapsulation. **a** Fluorescence images of WJMSC survival after 24 h culture, as tested by Live/Dead ^TM^ Cell Imaging Kit. Calcein staining (green) represents live cells while PI staining (red) represents dead cells. Scale bar, 400 μm. **b** Quantitation data of the percentage of dead cells per field. *n* = 3. **c** Quantitation data of relative cell viability in different groups after 24 h culture, as evaluated by CCK8 testing. *n* = 6. **d** Fluorescence images of WJMSC proliferation after 24 h culture, as tested by Cell Light ^TM^ EdU Kit. EdU positive (green) represents proliferating cells. Scale bar, 400 μm. **e** Quantitation data of the percentage of proliferating cells per field. *n* = 3. In **b**, **c**, and **e**, data were presented as mean ± SD. Statistical analyses were performed by one-way ANOVA followed by Tukey’s post test. ***p < 0.001; ns, no significance

To investigate whether PF-127 hydrogel was suitable for WJMSC encapsulation and for the biological effect of SAP on WJMSCs, WJMSCs were co-cultured with hydrogel and SAP in vitro for 24 h, and cell survival and proliferation assays were performed. As showed in Fig. 2a and b, 15.80 ± 2.59% of WJMSCs were dead in PF-127 encapsulation when compared to 0.74 ± 0.34% in the DF-12 control group. Supplementation of 400 μM SAP greatly reduced cell death rate to 5.21 ± 1.73%. However, there was no statistically significant difference in the improvement of cell survival when the concentration of SAP was increased to 800 μM (5.28 ± 1.77%). Similarly, CCK8 testing also demonstrated a lower cell viability in PF-127 encapsulation (0.37 ± 0.02) in comparison with the DF-12 control group (0.92 ± 0.02), but cell survival was significantly enhanced on supplementation of 400 μM SAP (0.67 ± 0.16) (Fig. 2c). Further, we investigated whether WJMSCs were able to proliferate in PF-127 hydrogel, as presented in Fig. 2d and e, 1.70 ± 0.18% of the cells showed proliferation in PF-127 encapsulation as opposed to 23.24 ± 3.26% in the DF-12 control group. Furthermore, supplementation of 400 μM SAP (2.11 ± 0.45%) or 800 μM SAP (2.03 ± 0.45%) exhibited no enhancement of WJMSCs proliferation. These results, together, imply that supplementation of 400 μM SAP significantly improved WJMSC survival but not their proliferation in PF-127 encapsulation.

### PF-127 plus SAP combination facilitates WJMSC-mediated skin wound healing

**Fig. 3:**
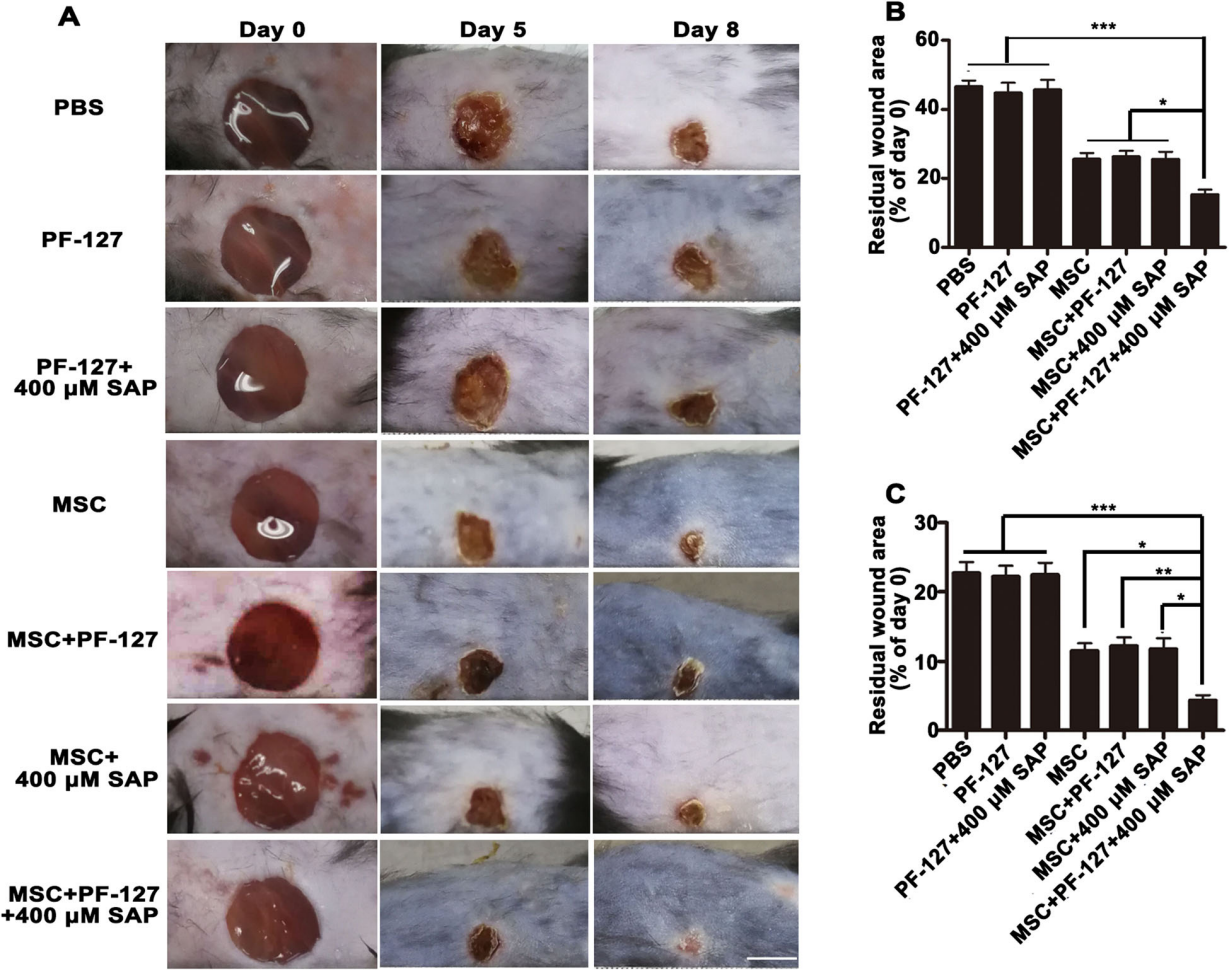
PF-127 plus SAP combination facilitates WJMSC-mediated wound healing. **a** Representative photographs of skin wound healing at day 5 and day 8 post-transplantation. Scale bar, 5000 μm. **b** Quantitation data of the percentage of residual wound area at day 5 post-transplantation (normalized to day 0). **c** Quantitation data of the percentage of residual wound area at day 8 post-transplantation (normalized to day 0). In **b** and **c**, data were presented as mean ± SD, *n* = 6. Statistical analyses were performed by one-way ANOVA followed by Tukey’s post test. *p < 0.05, **p < 0.01, ***p < 0.001

In order to investigate whether PF-127 plus SAP could facilitate WJMSC-mediated wound healing in vivo, this combination and others were topically transplanted onto excisional full-thickness wound bed. As shown in Fig. 3a and b, the wound in the WJMSC/PF127/SAP treatment group healed best at day 5 post-surgery compared to the other groups (PBS, 46.64 ± 4.09%; PF-127, 44.78 ± 6.69%; PF-127/SAP, 45.86 ± 6.71%; WJMSCs, 25.67 ± 3.51; WJMSCs/PF-127, 26.90 ± 3.72%; WJMSCs/SAP, 25.65 ± 4.11%; WJMSCs/PF-127/SAP, 16.04 ± 2.73%). Further, the wound in the WJMSCs/PF-127/SAP group almost completely healed at day 8 (4.34 ± 1.72%), whereas other groups still had visible unhealed wounds of variable sizes (PBS, 22.71 ± 3.55%; PF-127, 22.19 ± 3.50%; PF-127/SAP, 22.45 ± 3.89%; WJMSCs, 11.51 ± 2.45%; WJMSCs/PF-127, 12.22 ± 2.74%; WJMSCs/SAP, 11.78 ± 3.45%; Fig. 3a, c). Additionally, H&E staining (transverse cutting) also showed a smaller residual wound in the WJMSCs/PF-127/SAP group than that in other groups at day 8 post-surgery (Figures S2A and S2B). In summary, these results indicate that PF-127 plus SAP remarkably improved WJMSC-mediated full-thickness wound healing.

### PF-127 plus SAP combination promotes WJMSC-mediated dermis regeneration

**Fig. 4:**
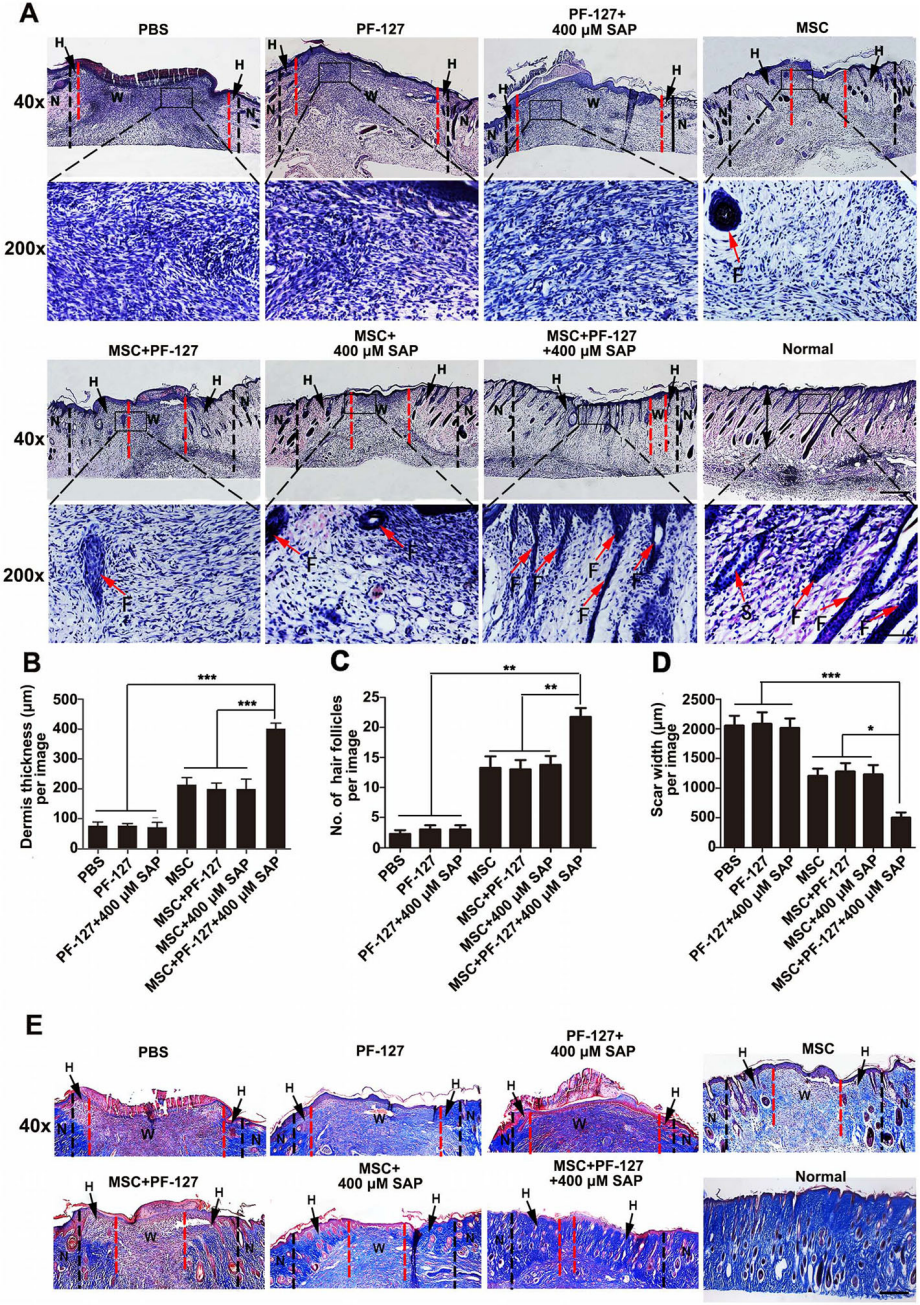
PF-127 plus SAP combination facilitates WJMSC-mediated dermis regeneration. **a** Hematoxylin-eosin (H&E) staining images of the wound bed and surrounding normal tissue (about 0.5 cm) at day 8 post-surgery. Scale bar: upper row, 500 μm; lower row, 100 μm. N, normal skin tissue, shown on both sides of the black imaginary line. H, healed skin tissue, shown between black and red imaginary lines. W, wound bed, unhealed skin tissue, shown between the red imaginary lines. F, hair follicle. **b** Quantitation data of healed dermis thickness at day 8 post-surgery. **c** Quantitation data of the number of newborn hair follicles at the healed site at day 8 post-surgery. **d** Quantitation data of scar width at the wound bed in different groups at day 8 post-surgery. In **b**, **c**, and **d**, data were presented as mean ± SD, *n* = 4. Statistical analyses were performed by one-way ANOVA followed by Tukey’s post-test. *p < 0.05, **p < 0.01, ***p < 0.001. **e** Masson’s trichrome staining images of the wound bed and surrounding normal tissue at day 8 post-surgery. Collagen fibers were stained dark blue. Scale bar: 500 μm. N, normal skin tissue, shown on both sides of the black imaginary line; H, healed skin tissue, shown between black and red imaginary lines; W, wound bed, unhealed skin tissue, shown between the red imaginary lines

To evaluate the therapeutic effect of PF-127 and SAP combination on WJMSC-mediated wound healing at the tissue level, we performed histological analysis on day 8 post-transplantation. The H&E staining (longitudinal cutting) shown in Fig. 4a suggested that the quality of dermis in the WJMSCs/PF-127/SAP group regenerated better as compared to the other groups, as demonstrated by increased dermis thickness in the WJMSCs/PF-127/SAP group (PBS, 76.29 ± 21.83 μm; PF-127, 76.73 ± 12.36 μm; PF-127/SAP, 72.00 ± 27.01 μm; WJMSCs, 213.52 ± 42.06 μm; WJMSCs/PF-127, 199.27 ± 35.09 μm; WJMSCs/SAP, 199.82 ± 56.89 μm; WJMSCs/PF-127/SAP, 401.33 ± 31.96 μm; Fig. 4b), augmented newborn hair follicles (PBS, 2.25 ± 1.09; PF-127, 3.00 ± 1.22; PF-127/SAP, 3.06 ± 1.24; WJMSCs, 13.25 ± 3.34; WJMSCs/PF-127, 13.00 ± 2.74; WJMSCs/SAP group, 13.75 ± 2.59; WJMSCs/PF-127/SAP group, 21.75 ± 2.59; Fig. 4 c), and decreased scar width (PBS, 2054.16 ± 286.70 μm; PF-127, 2082.30 ± 338.76 μm; PF-127/SAP, 2014.45 ± 274.94 μm; WJMSCs, 1277.33 ± 248.48 μm; WJMSCs/PF-127, 1202.59 ± 215.16 μm; WJMSCs/SAP, 1227.76 ± 274.10 μm; WJMSCs/PF-127/SAP, 498.22 ± 153.53 μm; Fig. 4d). Additionally, Masson’s trichrome staining demonstrated more collagen fibers deposited at the newly regenerated dermis in the WJMSCs/PF127/SAP group as compared to other groups (Fig. 4e).

### PF-127 plus SAP combination enhances WJMSCs migration in vitro and engraftment in the dermis in vivo

**Fig. 5:**
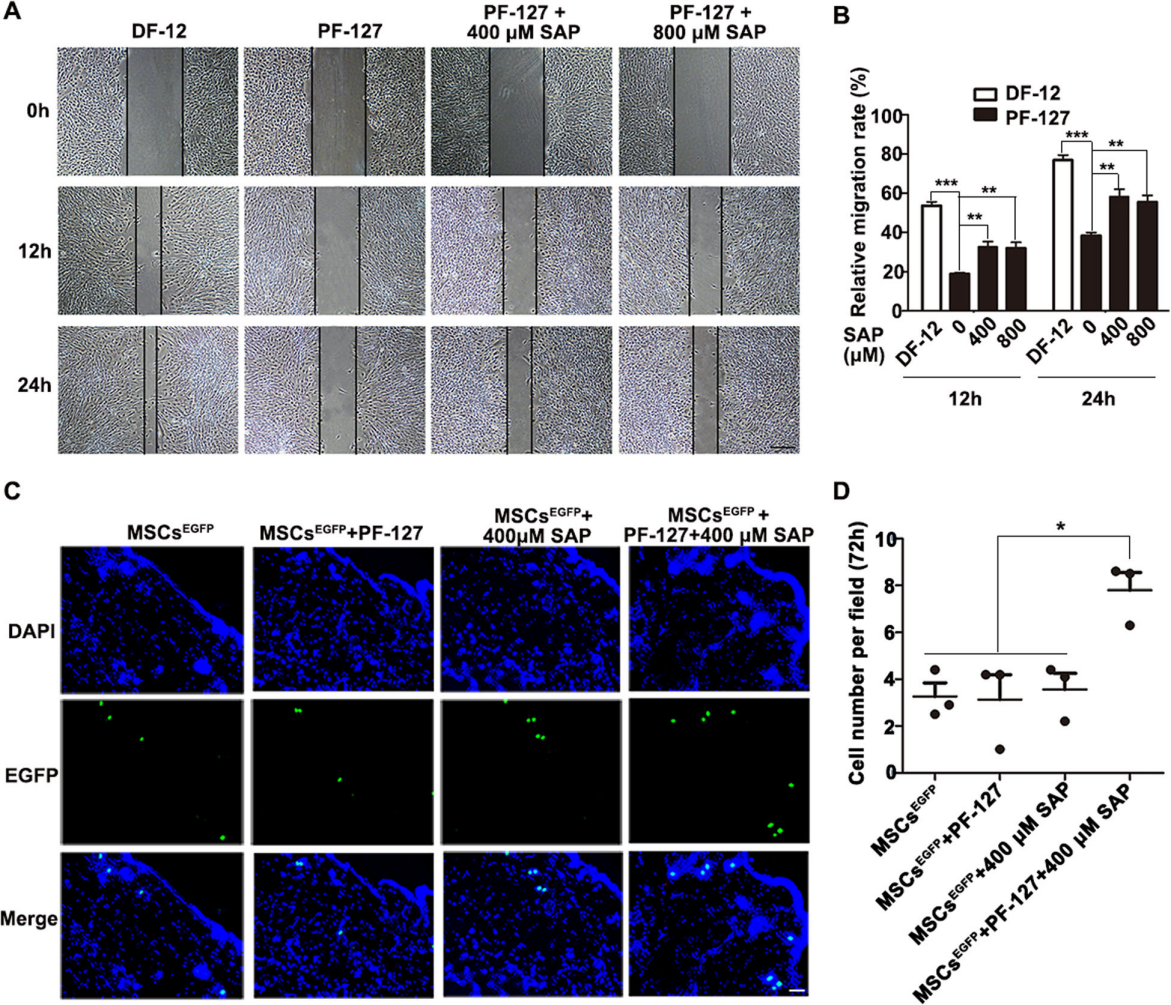
PF-127 plus SAP combination enhances WJMSCs migration in vitro and to the dermis in vivo. **a** Representative images of cell migration at 12 h and 24 h. Scale bar: 500 μm. **b** Quantitation data of relative cell migration rate at 12 h and 24 h, (normalized to 0 h). Data were presented as mean ± SD, *n* = 5. Statistical analyses were performed by one-way ANOVA analysis followed by Tukey’s post test. **p < 0.01, ***p < 0.001. **c** Representative fluorescence images of EGFP-overexpressing MJMSCs in different groups at 72 h post-transplantation, which was examined by cryo-sectioning. Signals: EGFP, green; DAPI, blue. Scale bar: 50 μm. **d** Quantitation data of cell number per field at 72 h in different groups. Data were presented as mean ± SD, *n* = 3. Statistical analyses were performed by one-way ANOVA analysis followed by Tukey’s post test. *p < 0.05

It is anticipated that cell engraftment in the target site is one of the most potentially therapeutic mechanisms of MSCs [35, 36]. Therefore, we hypothesized that PF-127 and SAP enhance WJMSCs retention and engraftment in the skin tissue, thereby improving its therapeutic effectiveness in wound healing. To evaluate this hypothesis, we first performed cell wound scratch assay in vitro to investigate whether PF-127 plus SAP promoted WJMSCs migration. As demonstrated in Fig. 5a and b, WJMSCs migration improved significantly when PF-127 was supplemented with 400 μM SAP (32.58 ± 5.44% at 12 h, 58.24 ± 7.57% at 24 h) or 800 μM SAP (31.90 ± 5.91% at 12 h, 55.44 ± 6.84% at 24 h) as opposed to the PF-127 group alone which showed slower cell migration (18.88 ± 1.32% at 12 h, 38.32 ± 3.19% at 24 h); however, the level of migration was lower in the WJMSCs/PF-127/SAP group when compared to that in the DF-12 control group (53.60 ± 3.89% at 12 h, 76.90 ± 5.16% at 24 h).

To further investigate whether PF-127 plus SAP exerted an enhanced effect on WJMSCs engraftment in the skin tissue following topical administration in vivo, we first established an EGFP-overexpressing stable cell line by transduction of WJMSCs with lentivirus vector encoding EGFP (Figures S3A and S3B). The transduced WJMSCs were referred to as MSCs^EGFP^. Next, MSC^EGFP^, MSC^EGFP^/PF-127, MSC^EGFP^/400 μM SAP, and MSC^EGFP^/PF-127/400 μM SAP (1 × 10^6^ cells per wound) were topically administered onto the site of the wound after surgery. The skin tissue was collected from each group at 24 h and 72 h post-injection and subjected to in situ immunofluorescence staining. We found that the quantity of WJMSCs accumulated in the dermis in the MSC^EGFP^/PF-127/400 μM SAP group was higher as compared to the other three groups, at both 24 h (MSC^EGFP^, 7.37 ± 2.07; MSC^EGFP^/PF-127, 7.78 ± 1.72; MSC^EGFP^/400 μM SAP, 7.40 ± 1.34; MSC^EGFP^/PF-127/400 μM SAP, 18.03 ± 2.72; Figures S3C and S3D) and 72 h (MSC^EGFP^, 3.27 ± 0.82; MSC^EGFP^/PF-127, 3.13 ± 1.51; MSC^EGFP^/400 μM SAP, 3.57 ± 0.57; MSC^EGFP^/PF-127/400 μM SAP, 7.80 ± 1.06; Fig. 5c, d). Conclusively, these results demonstrated that PF-127 plus SAP significantly enhanced WJMSCs migration and engraftment in the dermis in vivo.

### PF-127 plus SAP combination improves WJMSC-mediated M2 macrophage formation, cell proliferation, and angiogenesis

To study the potentially therapeutic mechanism of WJMSCs in wound healing, we carried out immunohistochemical analysis of CD163, Ki-67, and CD31 at day 8 post-transplantation.

**Fig. 6:**
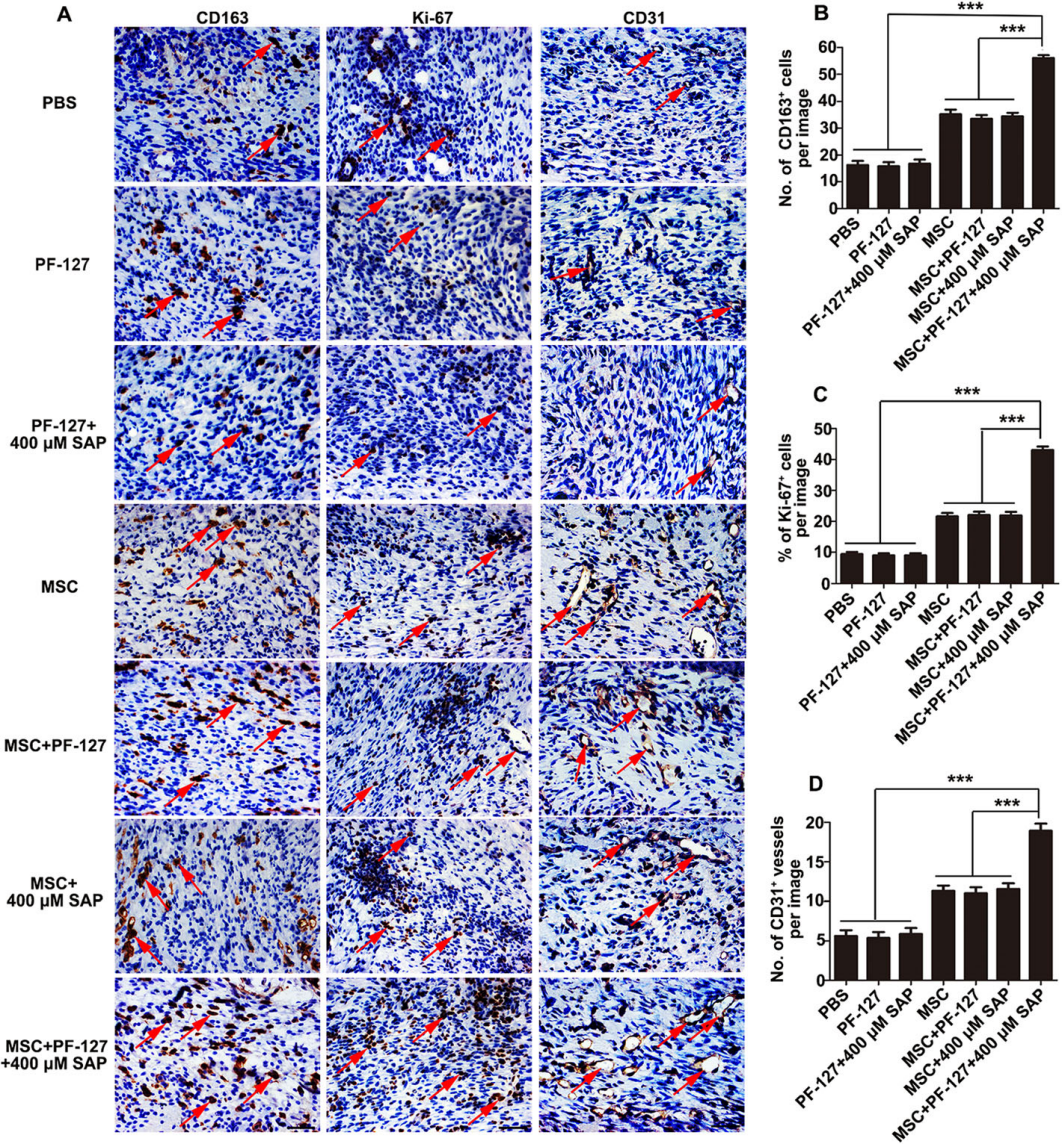
PF-127 plus SAP combination improves WJMSC-mediated M2 macrophage formation, cell proliferation, and angiogenesis. **a** Immunohistochemical staining images of CD163, Ki-67, and CD31 of the wound edge at day 8 post-surgery. Scale bar: 50 μm. **b** Quantitation data of the total number of CD163-positive M2 macrophages. **c** Quantitation data of the percentage of Ki-67-positive proliferating cells. **(D)** Quantitation data of the total number of CD31-positive newly developed blood vessels. In **b**, **c**, and **d**, data were presented as mean ± SD, *n* = 4. Statistical analyses were performed by one-way ANOVA followed by Tukey’s post test. ***p < 0.001

M2 macrophage, which is involved in the removal of pathogens and cell debris, secretion of anti-inflammatory factors, and synthesis of the ECM matrix, is thought to play a crucial role in the whole wound healing process [37, 38]. In this study, immunohistochemical staining of CD163 was conducted to evaluate M2 macrophage formation. As shown in Fig. 6a, newly formed M2 macrophages could be found in all the groups, but its number was highest in the WJMSCs/PF-127/SAP group (PBS, 16.26 ± 6.62; PF-127, 15.84 ± 6.29; PF-127/SAP, 16.79 ± 6.49; WJMSCs, 35.21 ± 7.38; WJMSCs/PF-127, 33.53 ± 5.95; WJMSCs/SAP, 34.42 ± 5.70; WJMSCs/PF-127/SAP, 56.05 ± 4.55; Fig. 6b). This result suggests that the enhancement of WJMSC-mediated wound healing by PF-127 and SAP is closely related to immunomodulatory effects.

Cellular proliferation of different cell types including endothelial cells, fibroblasts, keratinocytes, and macrophages that occurred at about days 3–10 after injury is supposed to promote granulation tissue formation, which is a biological marker in the process of wound healing [39]. In this study, immunohistochemical staining of Ki-67 was performed to determine total cellular proliferation. As shown in Fig. 6a and c, the level of Ki-67 positive cells in the WJMSCs/PF-127/SAP group was markedly higher as compared to other groups (PBS, 9.40 ± 3.07%; PF-127, 8.96 ± 3.02%; PF-127/SAP, 8.99 ± 3.10%; WJMSCs, 21.61 ± 4.72%; WJMSCs/PF-127, 22.01 ± 4.74%; WJMSCs/SAP, 21.90 ± 5.01%; WJMSCs/PF-127/SAP, 43.04 ± 5.24%). This result implied better granulation tissue regeneration in the WJMSCs/PF-127/SAP group.

Angiogenesis, a crucial event occurring during the entire wound healing process from the initial inflammation response stage until the end of the tissue remodeling stage, is involved in the restoration of blood flow and transportation of nutrients to the injured site [40]. In this study, immunohistochemical staining of CD31 was carried out to assess neovascularization. As demonstrated in Fig. 6a and d, the number of round or oval-shaped newly formed blood vessels in the WJMSCs/PF-127/SAP group was significantly higher than the other groups (PBS, 5.63 ± 2.83; PF-127, 5.38 ± 2.93; PF-127/SAP, 5.86 ± 2.98; WJMSCs, 11.31 ± 2.62; WJMSCs/PF-127, 11.02 ± 3.02; WJMSCs/SAP group, 11.56 ± 2.78; WJMSCs/PF-127/SAP, 18.94 ± 3.47). This result insinuated that the enhancement of WJMSCs-mediated wound healing by PF-127 and SAP may occur through stimulation of angiogenesis.

Considering all the results, we believe that PF-127 plus SAP is the best combination for the enhancement of WJMSCs engraftment in the dermis, which then contributes to the therapeutic effectiveness of WJMSCs in full-thickness cutaneous wound healing through M2 macrophage formation and angiogenesis.

## Discussion

In this study, we identified SAP as a new cell membrane-stabilizing agent that significantly improves WJMSCs survival in PF-127 encapsulation. Further, the combination of PF-127 and SAP exerted an enhanced effect on WJMSCs migration and engraftment in the dermis in vivo, which then contribute to its therapeutic effectiveness in full-thickness cutaneous wound healing through M2 macrophage formation and angiogenesis.

Recently, tissue engineering and regenerative medicine emerged as an important method to facilitate the regeneration of damaged tissue and have gained more popularity and attention [41]. These bioengineering technologies, which generally involve the application of a biomaterial, stem cells, and biologically active factors, have been extensively explored in skin soft tissue defects [42]. For example, it was reported that the combined use of hyaluronic acid and platelet-rich plasma (PRP) was effective in the regeneration of the lower-extremity wounds or the combined application of fat graft, adipose-derived stromal vascular fraction cells, and PRP was more effective in the correction of scars on the face than traditional scar surgical excision [43, 44]. In this study, we would like to investigate the therapeutic efficiency of the combination of PF-127, WJMSCs, and SAP on full-thickness skin wound healing.

WJMSCs are a promising candidate for cell therapy and tissue regeneration due to their beneficial characteristics such as easy isolation, self-renewal capacity, multipotency, and immunoregulatory effects [45]. However, their therapeutic efficacy needs to be further improved. It is believed that poor cell engraftment in the injured tissue is one of the obstacles limiting the therapeutic efficacy of stem cells [46]. Usually, the clinical application of stem cells is through intravenous injection; however, it was found that most of them got distributed in the lungs and only few cells successfully migrated to the site of injury [47, 48]. On the contrary, some researches demonstrated that locally transplanted MSCs could largely engraft in the target site [49, 50]. Therefore, in this study, to improve cell engraftment, we employed topical transplantation of WJMSCs onto the wound site.

Limited retention of sustained cells at the injected site due to cell suspension run-off after topical administration is anticipated to be another obstacle hampering stem cell therapeutic efficiency on wound regeneration. To overcome this problem, encapsulating cells in hydrogels in order to retain injected cells at the site of administration without run-off has been investigated [51, 52]. Murphy et al. demonstrated that transplantation of MSCs encapsulated in an engineered fibrin hydrogel could localize more cells at the affected site, which significantly promoted wound healing as compared to transplantation of cells alone [53]. Another study showed that mouse bone marrow mesenchymal stem cells (BMSCs) seeded in a composite scaffold based on arginine polymer and chitosan hydrogel maintained a high cell density and viability, and this combination effectively enhanced healing of severe burn wounds [54]. In our research, we used a thermo-reversible hydrogel PF-127 as WJMSCs encapsulation scaffold for the treatment of excisional full-thickness skin wound. The thermo-sensitive characteristic of the hydrogel allows it to promote cell attachment or retention at the site of transplantation [55]. Several studies have shown the therapeutic benefits of PF-127 as cell encapsulation scaffold in tissue engineering, and it has been used as scaffolds for porcine chondrocytes, somatic lung progenitor cells, and human adipose-derived stem cells [56–58]. However, it has also been reported that PF-127 encapsulation exerts a negative effect on cell viability, which is potentially destructive to cell-based therapy [23, 32]. Indeed, we demonstrated a 15.80 ± 2.59% decrease in cell survival at a concentration of 20% (w/v) PF-127 encapsulation after 24 h. Further, in vivo animal experiments displayed no statistically significant improvement on wound healing in the WJMSCs/PF-127 group as compared to the WJMSCs group. We proposed that these unsatisfactory results are closely related to PF-127 cytotoxicity. Therefore, before in vivo transplantation, it is important to develop a method to improve cell survival in PF-127 encapsulation.

It has been reported that several kinds of cell membrane-stabilizing agents such as hydrocortisone and vitamin C are added to the PF-127 gel formulations to increase cell viability [24, 32]. In this study, we proposed vitamin C analog SAP as a new effective cell membrane-stabilizing agent. SAP seems to be an optimal antioxidant candidate for skin tissue engineering applications because of its higher physical-chemical stability when compared to ascorbic acid which is easily oxidized in aqueous solution or on exposure to air [59]. Recent study has shown that SAP can act as an antioxidant to reduce facial skin sebum secretion in females [60]. Also, another study demonstrated the efficacy of SAP in the prevention and treatment of acne vulgaris, which is one of the most common inflammatory skin disorders, with no side effects [61]. In our study, both live and dead cell assay and CCK8 testing demonstrated that the addition of 400 μM SAP greatly enhanced WJMSCs viability in PF-127 encapsulation as compared to the PF-127 group alone in vitro after 24 h culture. Further, our in vivo assays revealed that PF-127 plus 400 μM SAP significantly facilitated WJMSC-mediated wound closure and dermis regeneration when compared to other groups at the 8th day post-transplantation; this was the first time beneficial effects of PF-127 and SAP composite on improving the effectiveness of WJMSC-mediated full-thickness wound healing were reported. It is believed that enhancement in local recruitment of cells and engraftment in the target tissue is one of the therapeutic mechanisms of MSCs [62, 63]. Thus, we propose that our in vivo wound healing results in the WJMSCs/PF-127/SAP group are attributed to more WJMSCs engraftment in the skin tissue aided by the effect of PF-127 in prolonging the in situ residence time of WJMSCs and the antioxidative effect of SAP in maintaining intracellular reactive oxygen species (ROS) homeostasis in PF-127 encapsulation. Indeed, we found that PF-127 plus 400 μM SAP significantly enhanced WJMSCs migration in vitro and promoted increased accumulation of WJMSCs in the dermis in vivo, which ultimately contributes to its effectiveness in full-thickness cutaneous wound healing through M2 macrophage formation and angiogenesis.

## Conclusion

The present study demonstrates that SAP can be used as a new cell membrane-stabilizing agent to improve cell survival in PF-127 encapsulation. Further, the PF-127 plus SAP combination enhances WJMSCs engraftment in the dermis, which then contributes to its therapeutic effectiveness in full-thickness cutaneous wound healing through potential M2 macrophage formation and angiogenesis.

## Supplementary information

**Additional file 1: Figure S1.** WJMSCs flow cytometry identification. (**A**) Flow cytometry analysis of P1 cells using mesenchymal stem cells markers (HLA-ABC, CD105, CD13, CD29, CD44, CD73), endothelial cells marker (CD31), hematopoietic cells markers (CD14, CD45), and MHC class II protein HLA-DR. Isotypic antibodies (IgG1-PE and IgG1-FITC) were used as negative controls. **Figure S2.** PF-127 plus SAP combination promotes WJMSCs-mediated wound healing. **(A)** Hematoxylin-eosin (H&E) staining images (transverse cutting) of the wound site together with surrounding normal skin tissue in different groups at day 8 after surgery. Scale bar: 500 μm. (**B**) Quantitation data of residual wound area at day 8 after surgery. Data were presented as mean ± SD, *n* = 4. Statistical analyses were performed by One-way ANOVA analysis followed by Tukey’s post-test. **p < 0.01, ***p < 0.001. **Figure S3.** PF-127 plus SAP combination promotes WJMSCs engraftment into dermis. (**A**) Construction of a WJMSC line stably expressing EGFP. **(B)** Western Blot confirmed EGFP protein in the WJMSC line. **(C)** Representative fluorescence images of EGFP-overexpressing WJMSCs in different groups at 24 h post-transplantation, which was examined by cryo-sectioning. Signals: EGFP, green; DAPI, blue. Scale bar: 50 μm. **(D)** Quantitation data of cell number per field at 24 h in different groups. Data were presented as mean ± SD, *n* = 3. Statistical analyses were performed by One-way ANOVA analysis followed by Tukey’s post-test. *p < 0.05.

## Data Availability

All data generated and/or analyzed in this study are included in this published article.

## Abbreviations

ANOVA: Analysis of variance
BMSCs: Bone marrow mesenchymal stromal cells
CCK8: Cell Counting Kit-8
CD: Cluster of differentiation
CFU-F: Fibroblast colony-forming unit
DAPI: Diamidinophenylindole
DMEM-F12: Dulbecco’s modified Eagle’s medium-F12
EDTA: Ethylenediaminetetraacetic acid
EdU: Ethynyldeoxyurid
ESCs: Embryonic stem cells
FBS: Fetal bovine serum
FITC: Fluorescein isothiocyanate
iPSCs: Induced pluripotent stem cells
PBS: Phosphate buffer saline
PE: Phycoerythrin
PF-127: Pluronic F-127
PFA: Paraformaldehyde
ROS: Reactive oxygen species
SAP: Sodium ascorbyl phosphate
UC: Umbilical cord
WJMSCs: Wharton’s jelly mesenchymal stem cells
