# Supplementary figures and images for: PF-127 hydrogel plus sodium ascorbyl phosphate improves Wharton’s jelly mesenchymal stem cell-mediated skin wound healing in mice
Source: Stem Cell Res Ther. 2020 Apr 3;11:143. doi: 10.1186/s13287-020-01638-2 (PMC7119174; doi:10.1186/s13287-020-01638-2)

# Supplement Figure 1

**A**

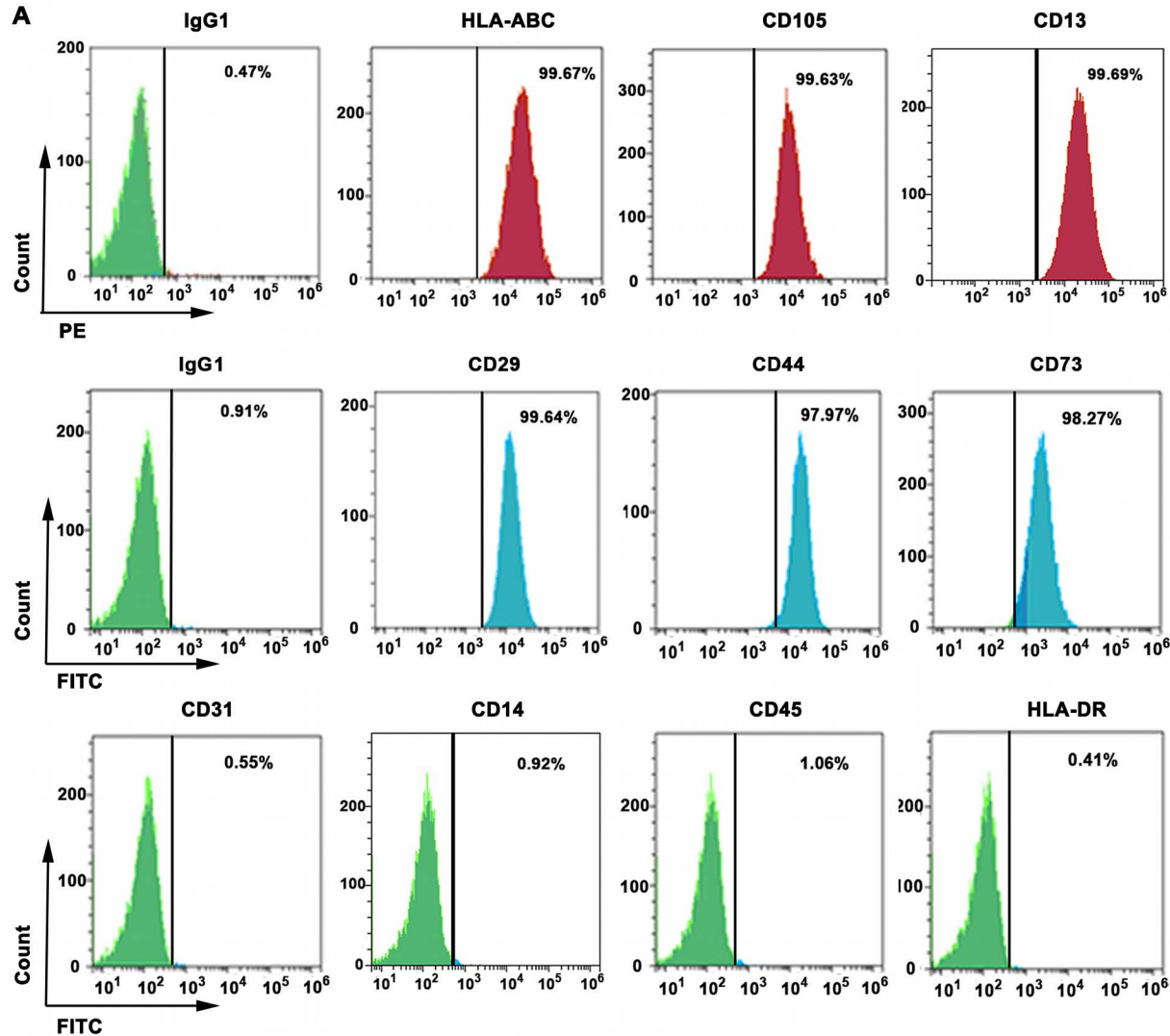

**Supplementary Figure 2**

**A**

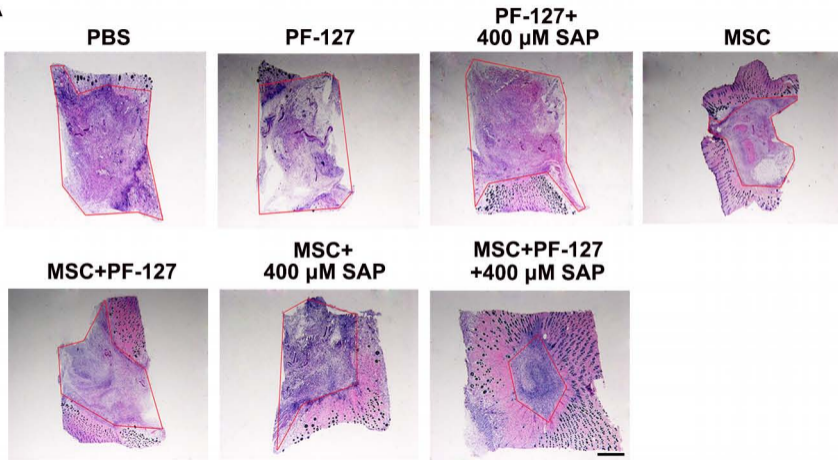

**B**

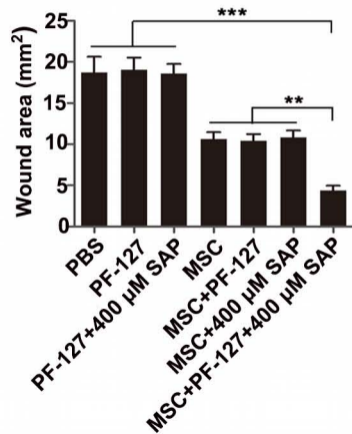

**Supplementary Figure 3**

**A**

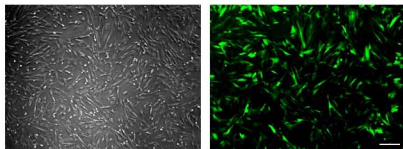

**B**

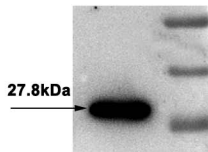

**C**

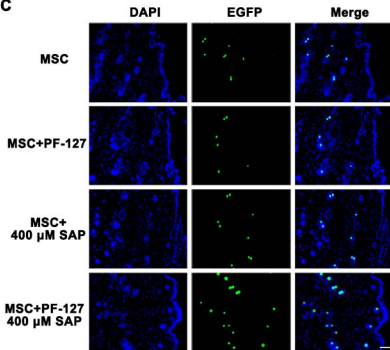

**D**

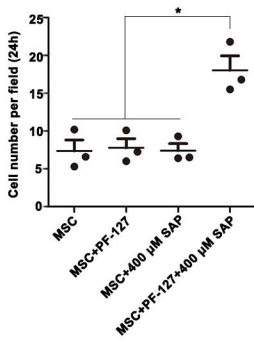

Supplement: Supplementary file 1 — Additional file 1: Figure S1. WJMSCs flow cytometry identification. (A) Flow cytometry analysis of P1 cells using mesenchymal stem cells markers (HLA-ABC, CD105, CD13, CD29, CD44, CD73), endothelial cells marker (CD31), hematopoietic cells markers (CD14, CD45), and MHC class II protein HLA-DR. Isotypic antibodies (IgG1-PE and IgG1-FITC) were used as negative controls. Figure S2. PF-127 plus SAP combination promotes WJMSCs-mediated wound healing. (A) Hematoxylin-eosin (H&E) staining images (transverse cutting) of the wound site together with surrounding normal skin tissue in different groups at day 8 after surgery. Scale bar: 500 μm. (B) Quantitation data of residual wound area at day 8 after surgery. Data were presented as mean ± SD, n = 4. Statistical analyses were performed by One-way ANOVA analysis followed by Tukey’s post-test. **p < 0.01, ***p < 0.001. Figure S3. PF-127 plus SAP combination promotes WJMSCs engraftment into dermis. (A) Construction of a WJMSC line stably expressing EGFP. (B) Western Blot confirmed EGFP protein in the WJMSC line. (C) Representative fluorescence images of EGFP-overexpressing WJMSCs in different groups at 24 h post-transplantation, which was examined by cryo-sectioning. Signals: EGFP, green; DAPI, blue. Scale bar: 50 μm. (D) Quantitation data of cell number per field at 24 h in different groups. Data were presented as mean ± SD, n = 3. Statistical analyses were performed by One-way ANOVA analysis followed by Tukey’s post-test. *p < 0.05. [file 13287_2020_1638_MOESM1_ESM.pdf]
